# Nuclear medicine imaging methods of early radiation-induced cardiotoxicity: a ten-year systematic review

**DOI:** 10.3389/fonc.2023.1240889

**Published:** 2023-10-09

**Authors:** Jordan Eber, Cyrille Blondet, Clara Le Fevre, Isabelle Chambrelant, Fabrice Hubele, Olivier Morel, Delphine Antoni, Georges Noel

**Affiliations:** ^1^ Institut de Cancérologie Strasbourg Europe (ICANS), Department of Radiation Oncology, Strasbourg, France; ^2^ Institut de Cancérologie Strasbourg Europe (ICANS), Department of Nuclear Medicine, Strasbourg, France; ^3^ Nouvel Hôpital Civil, Department of Cardiology, Strasbourg, France; ^4^ Strasbourg University, Institut Centre national de la recherche scientifique (CNRS), Institut Pluridisciplinaire Hubert Curien (IPHC) UMR 7178, Centre Paul Strauss, UNICANCER, Strasbourg, France

**Keywords:** radiotherapy, radiation-induced heart disease, positron emission tomography, single-photon emission computed tomography, nuclear medicine imaging

## Abstract

**Introduction:**

Radiotherapy has significantly improved cancer survival rates, but it also comes with certain unavoidable complications. Breast and thoracic irradiation, for instance, can unintentionally expose the heart to radiation, leading to damage at the cellular level within the myocardial structures. Detecting and monitoring radiation-induced heart disease early on is crucial, and several radionuclide imaging techniques have shown promise in this regard.

**Method:**

In this 10-year review, we aimed to identify nuclear medicine imaging modalities that can effectively detect early cardiotoxicity following radiation therapy. Through a systematic search on PubMed, we selected nineteen relevant studies based on predefined criteria.

**Results:**

The data suggest that incidental irradiation of the heart during breast or thoracic radiotherapy can cause early metabolic and perfusion changes. Nuclear imaging plays a prominent role in detecting these subclinical effects, which could potentially serve as predictors of late cardiac complications.

**Discussion:**

However, further studies with larger populations, longer follow-up periods, and specific heart dosimetric data are needed to better understand the relationship between early detection of cardiac abnormalities and radiation-induced heart disease.

## 1 Introduction

Radiotherapy (RT) has a major role in the treatment of breast and thoracic malignancies, improving survival (1). However, long-term cancer survivors face an increased risk of radiation-induced heart disease (RIHD). Thoracic RT can unintentionally expose the heart to radiation, leading to both early and late complications: myocardial disease, pericarditis, coronary artery disease (CAD), valvular heart disease, and conduction system dysfunction (2). RIHD is the most common nonmalignant cause of death among cancer survivors who undergo RT (3–7). To minimize RIHD, understanding the underlying pathophysiology is vital to reduce incidental heart irradiation and enable early diagnosis.

### 1.1 Myocardial perfusion imaging

Early alteration of cardiac perfusion has been described after RT and myocardial perfusion single photon computed tomography (MPS) was proposed as an accurate tool to detect and monitor RIHD. Several studies report evidence of early radiation-associated cardiac changes identified with MPS after adjuvant RT (8–10). MPS is a noninvasive diagnostic tool used in clinical practice for the assessment of cardiac perfusion defects (PDs) and enabled the characterization of key parameters, including ventricular volumes, left ventricular ejection fraction (LVEF) together with wall motion abnormalities. At rest or during stress (exercise or pharmacologic), a radiotracer is injected intravenously and is taken up by the myocardium in quantities proportional to myocardial perfusion. Radiotracers such as [^99m^Tc]-sestamibi or [^99m^Tc]-tetrofosmin are absorbed by healthy myocardial cells. Following RT, regions with reduced uptake indicate impaired myocardial blood flow (MBF), typically due to radiation-caused damage to the endothelium and capillary disturbances (11, 12).

Noninvasive myocardial perfusion imaging by positron emission tomography (PET) can be used to measure MBF and therefore detect perfusion abnormalities. Radiotracers such as rubidium 82, [^15^O]-H2O, [^13^N]-ammonia and [^18^F]-flurpiridaz are particularly useful for monitoring changes in MBF and myocardial flow reserve (MFR) (13, 14). These PET radiotracers have the capability to detect early markers of endothelial dysfunction, such as microvascular coronary disease and myocardial ischemia (15).

### 1.2 Myocardial metabolism

Fatty acid oxidation is the most efficient mode of myocardial energy production and requires a large amount of oxygen. Thus, alteration of fatty acid oxidation is a sensitive marker of ischemia and myocardial damage (16). In myocardial areas affected by decreased perfusion, fatty acids cannot be metabolized due to an oxygen deficiency, resulting in a low production of adenosine triphosphate. As the uptake of iodine-123 β-methyliodophenyl pentadecanoic acid ([^123^I]-BMIPP) is correlated with adenosine triphosphate; single photon emission computed tomography (SPECT) [^123^I]-BMIPP appears a reliable tool to detect decreased oxidative metabolism of free fatty acids as a surrogate marker of myocardial ischemia. While [^123^I]-BMIPP has only been used as an investigational agent, it shows promise in assessing both the severity and extent of myocardial damage (17, 18). Nevertheless, the BMIPP tracer’s availability is restricted due to its production complexities, regulatory challenges, and associated costs, limiting its widespread use in cardiac imaging.

Using a glucose analogue, [^18^F]‐fluorodeoxyglucose (^18^FDG) positron emission tomography ([^18^F]‐FDG-PET/CT) provides a unique assessment of myocardial metabolism by detecting changes in cardiomyocytes at an early stage before cardiotoxicity. Uptake of ^18^FDG correlates with changes in the cellular myocardial metabolism together with inflammation extent within the coronary arteries wall, preceding possible atherosclerotic plaque rupture and could also reflect macrophages content within the vulnerable plaque (19–21). Due to glucose’s role as an auxiliary energy source, PET examinations typically necessitate specific metabolic preparation in patients to achieve hyperinsulinemic euglycemia.

### 1.3 Linking imaging to pathophysiology

Understanding the techniques is pivotal, but the true value lies in correlating these findings with the pathophysiology of cardiotoxicity. Radiation affects the myocardium at the cellular level by causing DNA damage, oxidative stress, and apoptosis. Over time, these cellular disruptions lead to microvascular damage, impaired vasodilation, and metabolic shifts. Consequently, areas of reduced blood flow or increased glucose utilization mirror these pathophysiological changes, offering clinicians a tangible means to monitor, predict, and, ultimately, manage radiation-induced cardiotoxicity.

Although other imaging modalities such as magnetic resonance Imaging or X-ray computed tomography can explore myocardial perfusion, nuclear medicine constitutes the only modality to assess intimate myocardial cell metabolism. This systematic review focuses on nuclear medicine techniques to detect early cardiotoxicity after breast or thoracic radiation therapy.

## 2 Method

The systematic review was conducted according to the Preferred Reporting Items for Systematic Reviews and Meta-Analysis (PRISMA) guidelines (22). Database queries were performed from 1 January 2012 to 31 August 2022. Articles corresponding to the terms “((cardiac SPECT) or (positron emission tomography)) AND (heart) AND (“radiotherapy” OR “radiation therapy”)” were searched in the PubMed database (https://www.ncbi.nlm.nih.gov/pubmed). To be included in our systematic review studies had to meet specific inclusion criteria: i/patients had to have received breast or thoracic RT; ii/nuclear imaging was performed within the year after RT; and iii/the full text of the study was available in English. Exclusion criteria included the following: i/patients without a history of RT; ii/the use of non-human subjects; iii/case studies; iv/review articles; v/abstracts alone; and vi/editorials. Two reviewers (JE, CLF) independently screened all titles and abstracts. Disagreement between the two reviewers was resolved with discussion between them, or with a third reviewer (GN). From each article, the following data were collected: the number of patients, sex, age, primary cancer, RT modalities, nuclear imaging modalities, time interval between nuclear imaging and RT, cardiac dose, systemic cancer therapy, cardiac outcome, and nuclear imaging results.

**Figure 1:**
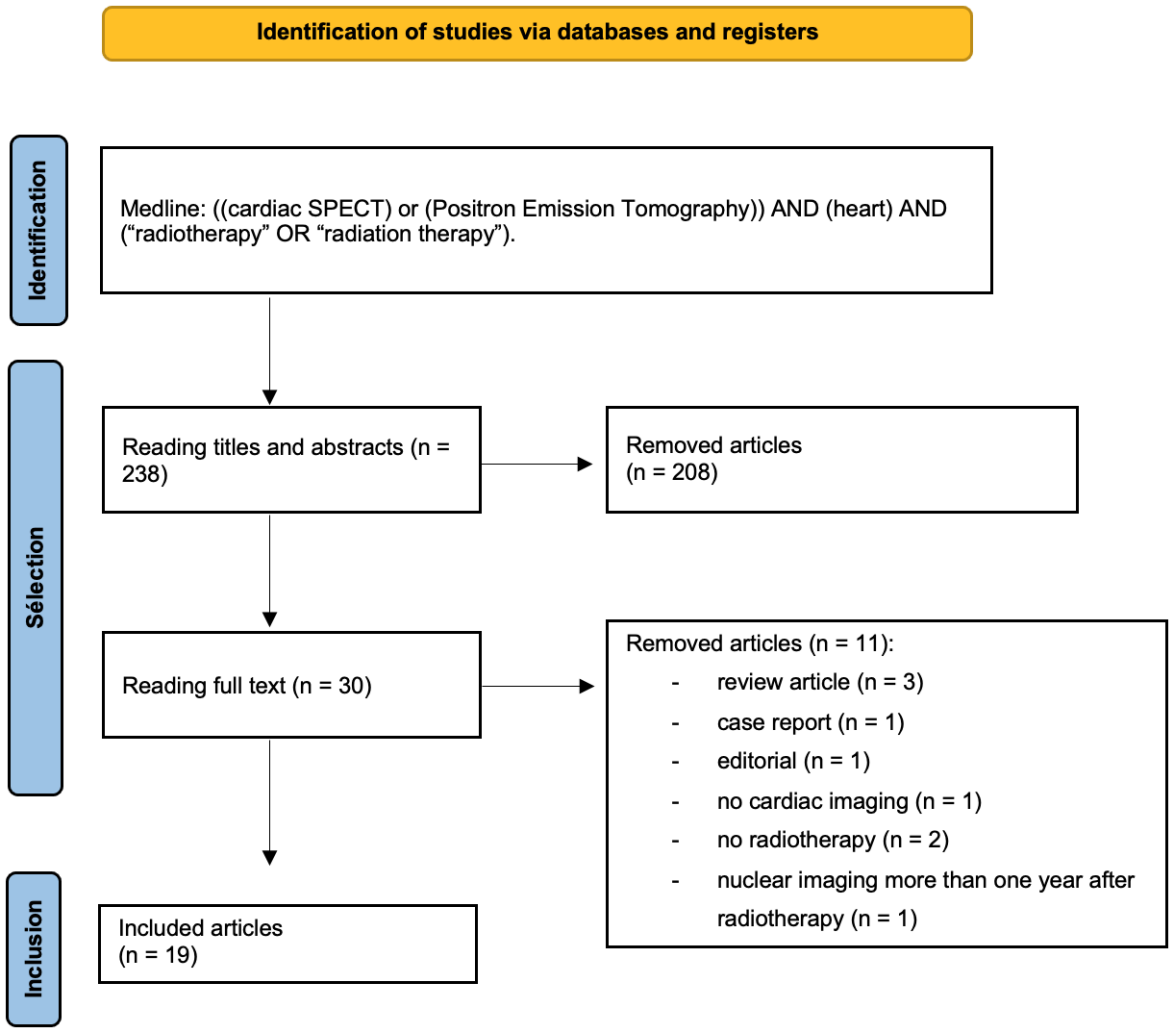
Flowchart of published selected articles.

Overall, 238 articles were retrieved. Among them, 219 articles were excluded because they did not meet the inclusion criteria. Ultimately, 19 studies were included. Among them, 11 evaluated radiation-induced myocardial metabolic dysfunctions (9 with PET, 2 with SPECT), and 8 evaluated radiation-induced myocardial perfusion dysfunctions (6 with SPECT and 1 with PET) (**Figure 1**).

## 3 Results

### 3.1 The myocardial perfusion single photon computed tomography

#### 3.1.1 Patient population

A total of 365 patients underwent MPS, among whom 347 were treated for breast cancer (BC) (232 left-sided breast cancer (LSBC)/115 right-sided breast cancer (RSBC)) and 18 for esophageal cancer. The sex ratio was 14 males and 351 females since most studies were about BC. The median age varied from 45.3 to 62.2 years old. Only one study reported performance status (PS), with PS < 2 and Karnofsky performance scale (KPS) > 70% as inclusion criteria (23). Cardiovascular risk factors varied among the patients: past smoker (40%-53%), active smoker (0%-67%), hypertension (19%-30%), diabetes mellitus (0%-11%), CAD (0%-5%), family history (0%-59%), and mean body mass index (28.0-29.2 kg/m²). Most breast cancer patients received chemotherapy, while two studies reported adjuvant hormonotherapy (67%-69%). Patients with esophageal cancer received concomitant cisplatin/5-fluorouracil (5-FU).

#### 3.1.2 MPS modalities

Five studies used [^99m^Tc]-sestamibi (23–27), with varied SPECT acquisition methods: four used rest acquisition (23, 24, 26, 28), one used adenosine stress (27), and one used rest and stress protocols (25). Perfusion assessments were conducted at different time points: one at six months post-RT without baseline SPECT (25), two at baseline and six months post-RT (26, 28) one at one year post-RT (27), one before and during irradiation (23), and one at multiple time points up to five years post-RT (24).

#### 3.1.3 Radiotherapy modalities

Prescribed radiation doses ranged from 40.05 Gray (Gy) to 50.00 Gy for breast/chest and lymph nodes, with one study using a 10 Gy boost. For esophageal cancer, the dose was 60–64 Gy. All studies used three-dimensional conformal radiation therapy (3DCRT), while three employed intensity-modulated radiotherapy (IMRT) (23, 24, 27). Cardiac-sparing techniques were used in three studies (26–28).

#### 3.1.4 MPS results

Four studies reported perfusion defects (PD) after RT, with rates ranging from 17% to 43% at 6 months for LSBC and 44% during RT for esophageal cancer (23–26). Wall motion abnormalities in the myocardium were reported in three studies, with two of them showing a significant decrease in wall motion post-therapy (23, 25). PD was mainly associated with cardiac dose, including LV dose-volume value and V_37Gy_–V_40Gy_ (23). No significant decrease in LVEF was found. Details of myocardial perfusion imaging results are in **Table 1**.

**Table 1:** Myocardial perfusion imaging.

Author	Design	Patient Age, y	Primitive	Imaging modalities; time interval	RT technique; [Prescribed dose]	Results
Chung et al., 2013 (27)	Prospective randomized controlled study	32 50	Breast	Pharmacological stress (adenosine) SPECT [^99m^Tc]-MIBI; Before RT and 1 year post RT	3DCRT or (n=14) or IMRT with ABC (n=18); [52.2 Gy in 1.74-Gy fx ± 10-Gy boost to the tumor bed or mastectomy scar]	No clinically significant PD were found after RT, with the average heart D_mean_ <5 Gy No correlations were found between cardiac doses and changes in PD, SSS, and LEVF
Zellar et al., 2014 (26)	Prospective randomized controlled study	43 58.7	Breast	Rest SPECT [^99m^Tc]-MIBI; Before and 6 months after RT	3DCRT with (n=28) or without (n=29) ABC; [40-48.6 Gy/1.8-2Gy fx (n=41) 40.05 Gy/2.7 Gy fx (n=2)]	PD in the apical segments of both ABC and No-ABC cohorts
Zhang et al., 2015 (23)	Prospective study	18 62.2	Esophageal (SCC)	Rest SPECT [^99m^Tc]-MIBI; Before and during RT	3DCRT (n=3) and IMRT (n=15); [60–64 Gy/30–32 fx]	Significant decreases in the wall motion, wall thickening and 45% had new myocardial PD. The V_37Gy_–V_40Gy_ was significantly higher (p < 0.05) in the patients with the new PD during RT.
Eftekhari et al., 2015 (25)	Prospective cohort study	71 45.3	Breast	Rest and stress (pharmacological and exercice) SPECT [^99m^Tc]-MIBI; No pre-RT, post-RT at 6 months	3DCRT; [46-50 Gy/23-25 fx]	PD in 42.9% (LSBC) and 16.7% (RSBC) (p = 0.02, Odds ratio = 1.46). No significant relationship between PD and % of the heart involved in the radiation field (p = 0.899)
Zagar et al., 2017 (28)	Prospective study	20 56	Breast	Rest SPECT (unkown-radiotracer); Before RT, post-RT at 6 months	3DCRT with DIBH; [42.72 Gy (2.67 Gy/16 fx), or 46-50 Gy (2 Gy/23-25 fx)]	0% had post-RT perfusion or wall motion abnormalities. 0% reported cardiac symptoms during RT or post-RT at 6 months.
Abraham et al., 2022 (24)	Randomized controlled trial	181 59	Breast	Rest SPECT [^99m^Tc]-MIBI; Before, 6 months, 1 year, 2 years, and 5 years post-RT	3DCRT (52%) and IMRT or tomotherapy (48%); [50 Gy/25 fx over 5 weeks]	RT was associated with short-term PD that improved within 1 year and was not correlated with late cardiac events. Ventricular V_5Gy_ and V_10Gy_ were correlated with late cardiac events. Cardiac events grade ≥ 2: 17.2% (LSBC)/5.5% (RSBC) (p = 0.024).
Żyromska et al., 2018 (29)	Prospective pilot study	15	Breast	Rest and stress [^15^O]-H_2_O PET/CT; Before RT and 2 and 8 months after RT	3DCRT; [42.5-45.0 Gy at 2.5-2.25 Gy/fx ± 10-11.5 Gy boost to the tumor bed or mastectomy scar]	Post-RT, 53% had reduced MBF, and 33% had increased MBF in both LSBC and RSBC. Persistent PD was noted in LADA-supplied segments at 2 and 6 months (p=0.018, p=0.032), along with decreased overall heart perfusion (p=0.036). Minimal radiation dose to the LADA correlated with MBF changes at 2 months (p=0.032), but no correlation was found with clinical heart toxicity symptoms.

[^99m^Tc]-MIBI, ^99m^Tc-sestamibi; 3DCRT, three-dimensional conformal radiation therapy; ABC, active breathing control; DIBH, deep inspiration breath hold; D_mean:_ mean dose; fx, fraction; Gy, Gray; IMRT, intensity-modulated radiotherapy; LADA, left anterior descending artery; LSBC, left sided breast cancer; MBF, myocardial blood flow; PD, perfusion defects; PET/CT, positron emission tomography/computed tomography; RSBC, right sided breast cancer; RT, radiotherapy; SCC, squamous cell carcinoma; SPECT, single-photon emission computed tomography; SSS, summed stress score.

#### 3.1.5 Clinical outcome

Two studies did not report clinical outcomes (26, 27). Abraham et al. observed ≥2 cardiac events, at a median follow-up of 127 months, among 20 out of 181 BC patients who received adjuvant RT, but did not specify if they were symptomatic (24). Another study noted increased heart rate and decreased R-R interval on the electrocardiogram (ECG) during RT without clinical symptoms (23). Some studies found no cardiac symptoms during the 6-month follow-up (25, 28).

### 3.2 Myocardial perfusion PET imaging

#### 3.2.1 Patient population

One study assessed perfusion PET imaging in 15 females with BC (6 RSBC/9 LSBC), aged 32-68 years (29). Four patients had cardiac comorbidities (diabetes or hypertension) but no cardiovascular disease history. Among them, 53% received pre-RT chemotherapy and 73% received post-RT hormonal therapy.

#### 3.2.2 PET modalities

Perfusion PET imaging was performed pre-irradiation and at 2- and 8-months post-RT. Resting and pharmacological stress with adenosine [^15^O]-H_2_O PET imaging were conducted for each patient.

#### 3.2.3 Radiotherapy modalities

All patients received tangential 3DCRT to the breast (n = 10) or chest wall (n = 5) with standard total doses of 42.5 or 45.0 Gy at 2.5 or 2.25 Gy per fraction, respectively. Patients with intact breasts (n = 10) received an additional boost of 11.25 or 10 Gy at 2.25 or 2.5 Gy per fraction to the tumor bed, respectively. Cardiac doses were not reported.

#### 3.2.4 PET results

MBF significantly changed two months after breast/chest wall irradiation, with a decrease in 53% of cases and an increase in 33% of cases. Stress testing was more sensitive, showing decreased perfusion in left anterior descending artery (LADA) supplied segments that persisted at 6 months. Minimal radiation dose to LADA correlated with MBF changes. Myocardial perfusion imaging results are reported in **Table 1**.

#### 3.2.5 Clinical outcome

The author did not find any correlation between radiological findings and cardiac symptoms at the 8-month follow-up.

### 3.3 Myocardial metabolic SPECT imaging

#### 3.3.1 Patient population

Two studies assessed myocardial metabolism using SPECT in esophageal cancer patients (30, 31). A total of 17 patients (5 + 12) were included, with median ages of 64.4 and 75.0 years. The male-to-female ratio was 14:3. Cardiac comorbidities varied, with active smoking in 67-100%, hyperlipidemia in 8-20%, hypertension in 17-20%, and diabetes mellitus in 0-8% of patients. None had a history of heart disease. Sixteen patients received concurrent chemotherapy with cisplatin/nedaplatin and 5-FU.

#### 3.3.2 SPECT modalities

Both studies used scintigraphy with [^123^I]-BMIPP. One study performed SPECT before irradiation and 6 months after RT (30) while other study evaluated patients prechemoradiotherapy (CRT), at preboost irradiation during CRT, at 3 months post-CRT, and 1 year post-CRT (31).

#### 3.3.3 Radiotherapy modalities

The prescribed radiation doses in both studies ranged from 60 to 66 Gy, including a dose of 40 Gy to the primary tumor and lymph nodes, and a boost of 20 to 26 Gy to the primary tumor and metastatic lymph nodes. The radiotherapy technique employed was 3DCRT, using anterior-posterior and oblique fields.

#### 3.3.4 SPECT results

Umezawa et al. reported dose-related myocardial metabolic disorder at 6 months after radiotherapy, with mean decreases of 8.7% to 19.0% in different dose regions (30). The other study found significant correlations between myocardial [^123^I]-BMIPP uptake and heart/LV dose at preboost and three months post-RT, but not at one year after CRT (31). Myocardial metabolic imaging results are provided in **Table 2**.

**Table 2:** Myocardial metabolic imaging.

Author	Design	Patient Age, y	Primitive	Imaging modalities; Imaging time interval	RT technique; [Prescribed dose]	Results
Konski et al., 2012 (32)	Retrospective	74 62	Esophageal (65 ADK, 0 SCC)	[^18^F]-FDG-PET/CT, no control diet, fasting > 4 h; Before RT and after with a median interval of 25 (10–76) days	3DCRT; [50.4 Gy (45 – 57.6 Gy) in 1.8 Gy fx]	Lateral myocardial wall and combined wall SUV_max_ decreased notably between pre- and post-CRT evaluations (p=0.009, p=0.035). No correlation between post-treatment myocardial FDG uptake changes and cardiac toxicity.
Evans et al., 2013 (33)	Retrospective	39 69	Lung	[^18^F]-FDG-PET/CT, no control diet, fasting > 6 h and glucose < 200 mg/dL; Before and post SBRT (median 6 and 17 months)	SBRT; [50 Gy in 4 fx]	23% had increased cardiac FDG uptake at heart V_20Gy_. In patients with ≥5 cm³ of the heart exposed to 20 Gy, 47% had increased FDG uptake, compared to 0% with <5 cm³ exposure.
Unal et al., 2013 (34)	Retrospective	38 60	Thoracic (lung (89.5%), esophageal (5.3%), Multiple myeloma (2.6%), Gastric (2.6%)	[^18^F]-FDG-PET/CT, no control diet, fasting > 6 h and glucose < 140 mg/dL; Before RT and after with a median time of 7.5 months (4–39 months)	3DCRT; [64 Gy (30–76 Gy)]	Visual analysis showed 74% regional, 13% diffuse, and 13% no significant myocardial FDG uptake. Patients with regional uptake had higher SUV values in irradiated segments compared to non-irradiated (p<0.001). No correlation was found between radiotherapy doses and SUV measurements or ratios in irradiated myocardium.
Ishida et al., 2018 (35)	Retrospective	41 66	Esophageal (SCC)	[^18^F]-FDG-PET/CT, no control diet, fasting > 4 h and glucose < 150 mg/dL; Only 4 had baseline PET, median time from the initial day of CRT to the PET was 11 months (7–21 months)	3DCRT; [60 Gy (50.4–60.0 Gy)]	In the ≥18-h fasting group, FDG accumulation decreased more (18% vs 71%, p=0.002) with a higher focal accumulation rate (65% vs 13%, p=0.001). Higher LV dose areas had increased SUV_max_ values (p<0.001).
Jo et al., 2020 (36)	Retrospective	103 51.2 (LSBC)/ 51.4 (RSBC)	Breast (55 LSBC; 48 RSBC)	[^18^F]-FDG-PET/CT, no control diet, fasting > 6 h and glucose < 150mg/dL; PET1 and curative surgical resection: 5 days PET 2 and the end of adjuvant CT: 13 days PET 3 and end of RT: 132 days PET 4 and end of RT: 353 days	3DCRT; [50.4 Gy/28 fx ± 10-16 Gy/5-8 fx boost dose to the tumor bed]	FDG uptake varied in LSBC post-3DCRT with >30 Gy, persisting at one-year follow-up, and significantly correlated with myocardial radiation dose. No change noted between LSBC and RSBC.
Vinogradsky et al., 2021 (37)	Data from prospective cohort	39 64	Lung (32 NSCLC; 7 SCLC)	[^18^F]-FDG-PET/CT, fasting > 18h, blood glucose level < 150 mg/dl; Baseline (1–2 weeks before RT) and post-RT (2–3 months after RT)	3DCRT (n = 6)/IMRT (n = 18); [60 Gy in 2Gy/fx]	In patients studied, FDG uptake increased in 5 (16.68% SUVR), decreased in 13 (-41.38% SUVR), and remained stable in 6 (-5.53% SUVR). Segments exposed to 20-30 Gy saw a 7.24% uptake increase post-RT.
Cho et al., 2022 (38)	Retrospective	133 67	Lung (93 SCC; 28 ADK; 12 others)	[^18^F]-FDG-PET/CT, no control diet, fasting >4 h and glucose < 180mg/dL; 11 (1–91) days after CRT	83% 3DCRT, 14% IMRT, 3% both; [60-66 Gy in 2.0, 2.2 or 2.4 Gy/fx during 5-6 weeks]	High myocardial FDG uptake is linked to cardiac events, particularly in patients with higher MHD. 32% experienced these events within a median of 36 months post-CRT.
Zakem et al., 2022 (39)	Retrospective	51 67	Esophageal (38 ADK; 13 SCC)	[^18^F]-FDG-PET/CT, no control diet; Before RT and 56 (3-692) days after RT	IMRT, 3DCRT/ [50.4 Gy/25 fx]	Cardiac SUV rises by 0.044 per 10 Gy dose increment. Disease stage and heart SUV_mean_ change significantly predict OS.
Sha et al., 2020 (40)	Retrospective	24 61	Esophageal (SCC)	[^18^F]-FDG-PET/CT, no control diet; Before RT and 97 11–477) days after RT	IMRT; [45 Gy- 60 Gy in 1.5 to 2 Gy/fx in 25–30 fx]	1.7% SUV_mean_ rise per 10 Gy, significantly affecting OS (HR 0.541, 95% CI 0.312–0.937). Living patients saw a 17.2% increase in cardiac SUV_mean_, whereas deceased patients had a 13.5% decrease (p = 0.048).
Umezawa et al., 2015 (30)	Pilot study	5 75	Esophageal	[^123^I]-BMIPP SPECT; Before RT and 6 months after	3DCRT; [60-66 Gy]	All patients experienced reduced uptake in areas exposed to RT. A trend in dose-effect relations for this decrease was noted at 6 months post-RT.
Takanami et al., 2016 (31)	Prospective study	12 63.4	Esophageal	[^123^I]-BMIPP SPECT; Pre-CRT, pre-boost irradiation during CRT, 3-months post-CRT, and 1-year post-CRT	3DCRT; [60–66 Gy]	At pre-boost, BMIPP uptake was linked with certain dose metrics but lost significance at 1-year post-CRT, with no correlation to mean dose or LV/heart V_20Gy_ at any stage.

[^123^I]-BMIPP SPECT, iodine-123 β-methyliodophenyl pentadecanoic acid single-photon emission computed tomography; [^18^F]-FDG-PET/CT, [^18^F]‐fluorodeoxyglucose positron emission tomography/computed tomography; 3DCRT, three-dimensional conformal radiation therapy; ABC, active breathing control; ADK, adenocarcinoma; CRT, chemoradiotherapy; fx, fraction; Gy, Gray; IMRT, intensity-modulated radiotherapy; LSBC, left sided breast cancer; LV, left ventricular; MHD, mean heart dose; ns, not served; NSCLC, non-small cell lung cancer; OS, overall survival; RSBC, right sided breast cancer; RT, radiotherapy; SBRT, stereotactic body radiation therapy; SCC, squamous cell carcinoma; SCLC, small cell lung cancer; SPECT, single-photon emission computed tomography; SUV _mean_, mean standardized uptake value; SUV, standardized uptake value; SUV_max_, maximum standardized uptake value; SUVR, standardized uptake value ratio.

#### 3.3.5 Clinical outcome

Both studies assessed brain natriuretic peptide (BNP) level, ECG, and pericardial effusion in all patients after RT. None of the 17 patients exhibited clinical symptoms. One study showed no significant change in BNP level (31), while the other reported a non-significant increase from 26.32 pg/ml to 58.44 pg/ml (30). ECG changes were observed in five out of 12 patient, and asymptomatic pericardial effusion was detected in seven out of twelve patients at 3 months post-CRT, and in three out of five patients at 6 months post-CRT (31).

### 3.4 Myocardial metabolic PET imaging

#### 3.4.1 Patient population

A total of 542 patients (333 males and 209 females) underwent PET/CT evaluation early after RT. The median age ranged from 45.3 to 62.2 years. The primary cancers included thoracic malignancies (34), esophageal cancer (32, 35, 39, 40), lung cancer (33, 37, 38), and BC (36). The KPS was reported in two studies, with median values of 80% (39) and 90% (37). The ECOG status was reported in one study, with 69% of patients classified as ECOG 1 and 31% as ECOG 2 (35). Cardiovascular risk factors were reported in seven studies, including active smoking (14% to 97%), diabetes mellitus (15% to 23%), dyslipidemia (4% to 41%), hypertension (15% to 56%), and preexisting cardiac disease (3% to 57%). Most patients received concomitant or adjuvant systemic therapies.

#### 3.4.2 PET modalities

All studies used [^18^F]‐FDG-PET/CT without specific preparation for cardiologic evaluation. Preparation methods typically involved fasting for >4 or 6 hours and a threshold glucose dose. The time interval between RT and PET ranged from 11 days to 17 months.

#### 3.4.3 Radiotherapy modalities

Four studies used IMRT (37–40), one study evaluated stereotactic body radiation therapy (SBRT) for lung cancer (33), and seven studies used 3DCRT (32, 34–36, 38, 40). Prescribed doses varied, ranging from 50.4 to 66.0 Gy for esophageal cancer, 50.4 Gy in 28 fractions with a boost dose of 10–16 Gy in 5–8 fractions for breast cancer, and a median dose of 64 Gy (30–76 Gy) for thoracic malignancies (34). In lung cancer, the median dose was 60 Gy (45-60 Gy) in 30 fractions with doses per fraction ranging from 1.5 to 2 Gy. The prescribed dose for SBRT was 50 Gy delivered in 4 fractions. Cardiac doses were reported in six studies (32, 33, 37–40), with MHD ranging from 8.3 Gy to 18.0 Gy.

#### 3.4.4 PET findings

All studies observed changes in FDG intake after RT, with most showing an increase in myocardial standardized uptake value (SUV) (33–40). However, three studies reported both FDG increase and decrease (37, 39, 40), and one study found only FDG decrease (32). Cho et al. found intense visual LV uptake in 46% of patients, moderate uptake in 34%, and mild uptake in 20%, with focal uptake observed in 7% of patients (38). Sha et al. reported that in a population of 24 patients irradiated for esophageal cancer, the average SUV ratio (SUVR) increased in five patients, decreased in 13 patients, and did not change significantly in six patients (40). SUV uptake appeared to be correlated with dose, with an average increase of 1.7% 37 or 0.044 SUV for every 10 Gy dose bin (39). Jo et al. found that for LSBC patients receiving adjuvant 3DCRT, FDG uptake in the myocardium irradiated with more than 30 Gy was significantly increased on both the one-year follow-up PET/CT and the post-RT PET/CT (36). Myocardial metabolic imaging results are reported in **Table 2**.

#### 3.4.5 Clinical outcome

Two studies found a significant correlation between pre- and post-RT SUV_mean_ changes and overall survival (37, 39). Three studies reported no cardiac symptoms during follow-up periods of 3 months, 7.5 months, and 11 months (34, 35, 40). Konski et al. identified 12 patients with treatment-related cardiac toxicity, with a median time to any cardiac toxicity of 4.2 months and to symptomatic cardiac toxicity of 8.3 months (32).

## 4 Discussion

### 4.1 Radiation-induced myocardial perfusion dysfunction

Radiotherapy can damage myocardial endothelial cells, leading to various cardiovascular complications such as coagulation activation, capillary disruption, swelling, thrombotic obstruction, impaired vasodilation, focal ischemia, and perivascular fibrosis (33, 39, 40). SPECT is a valuable diagnostic tool for detecting myocardial PD after RT. While the data on PD rates present inconsistencies, these can be primarily attributed to variations in MPS protocols and the timing of perfusion evaluations. Additionally, differences in baseline cardiovascular risk factors and individualized radiotherapeutic dose-volume specifications can further contribute to these discrepancies. Notably, only a single study implemented a rest/stress protocol (25). MPS not only discerns between reversible PD (indicative of ischemia) and irreversible PD (signifying infarction) but also sheds light on the underlying mechanisms driving radiation-induced coronary artery disease. Typically, rest PD is suggestive of myocardial fibrosis or degeneration. On the other hand, stress-induced perfusion irregularities, if not evident during rest, pinpoint endothelial dysfunction and related vasculopathies, elevating the risk of acute coronary syndrome (41).

A study on BC patients found that [^15^O]-H_2_O PET/CT effectively analyzed early subclinical changes in heart perfusion (29). However, Rasmussen et al. evaluated 20 women who received adjuvant RT for LSBC and did not observe any differences in rest or stress MBF between the irradiated and non-irradiated myocardium. The study’s limitations included a small sample size, lack of baseline PET/CT imaging, and absence of a control group (42).

### 4.2 Radiation-induced myocardial metabolic dysfunction

Radiation-induced damage can affect myocardial perfusion and metabolic function, leading to changes in energy sources and metabolism (43, 44). High-dose irradiation can impair mitochondrial function and shift energy production to anaerobic metabolism and glycolysis (45, 46). This altered metabolism can be detected through abnormal FDG uptake (47, 48) and decreased accumulation of [^123^I]-BMIPP. While most studies indicate an increase in myocardial FDG uptake, it is essential to acknowledge that some research points to a reduced FDG uptake in the myocardium (37, 39, 40). The shift in SUV values seems to correlate with patient outcomes: there is an observed increase in survivors and a decrease in non-survivors (37, 39). From a physiological standpoint, this can be understood as follows: radiation can induce cardiac microvascular damage, which escalates with the dose, consequently causing localized ischemia. The heart, in response to this irradiation, undergoes metabolic shifts or heightens its glucose uptake (leading to an increase in SUV) within the irradiated regions as a coping mechanism. Myocardium that cannot adapt, either due to pre-existing conditions or because the radiation damage exceeds a critical limit, is ultimately compromised, leading to a decline in SUV.

Standardized PET preparation protocols for evaluating myocardial damage in the irradiated myocardium are lacking. Differences in fasting and imaging protocols exist between [^18^F]‐FDG-PET/CT oncologic applications and cardiac inflammatory imaging applications (49). Various methods for detecting metabolic changes in the myocardium have been reported, but no specific cardiac preparation was used in the reviewed studies. Variations in FDG accumulation make it challenging to distinguish abnormal from physiological myocardial uptake (50). Despite the reported preparations, no specific cardiac preparation was used in the studies included in this review.

### 4.3 Dose relation

Previous studies have shown that PD occurs in the irradiation field and is correlated with cardiac exposure (8, 9). One study found new PD in the LADA region six months after irradiation, with severity linked to the LV volume receiving over 25 Gy. No perfusion changes were observed in other coronary arteries (51). This review confirms the consistent pattern and location of new PD in the radiation field and degree of cardiac exposure. In BC irradiation, particularly LSBC, the apex is commonly affected, and the LADA segment is the most impacted (23, 24, 26). Both SPECT [^123^I]-BMIPP studies showed early myocardial metabolic disorder after high-dose irradiation, but at 1-year post-CRT, there was no association between myocardial metabolic impairment and radiation dose. This suggests that other factors such as cardiovascular risk factors or chemotherapy may influence long-term myocardial metabolism, making it challenging to predict long-term radiation-induced heart disease based solely on radiation dose.

Previous literature on metabolic PET imaging found dose-response relationships in head and neck cancer (52) and lung cancer (53). Similarly, in this review, several studies reported dose-related FDG changes (33, 35–40). Evans et al. showed a correlation between heart tissue exposure to 20 Gy and cardiac FDG uptake (33). Jo et al. showed that for patients with LSBC, the increase in FDG uptake in the irradiated myocardium was significantly associated with the radiation dose to the myocardium and was persistently observed on the one-year follow-up PET/CT, suggesting that the damage to the myocardium was related to the radiation dose and was not a transitional phenomenon (36). Among advanced-stage lung cancer patients, Vinogradskiy et al. showed increasing FDG uptake in the heart as a function of the dose (37).

#### 4.3.1 Cardiac sparing

The impact of different techniques on preventing new PD is still controversial. Zellers et al. found no benefit of DBIH in relation to PD occurrence, but the heart was not consistently excluded from the RT field (26). Conversely, Zagar et al. showed no PD or wall motion abnormalities at six months with the use of DIBH, although the study had limitations such as SPECT being performed only at rest and a small sample size (28). IMRT is a potential solution for reducing high-dose irradiation to the myocardium, but the potential cardiac effects of low-dose mediastinal RT should also be considered. Notably, even a minimal radiation dose applied to the LADA may predict radiation-induced heart toxicity, suggesting the absence of a threshold dose (7).

#### 4.3.2 Clinical outcome

Stress/rest MPS is a sensitive examination used for monitoring and detecting cardiac diseases (54, 55). It can detect early abnormalities after RT, but its predictive value for long-term cardiac events is still uncertain (56–58). While some studies have found associations between PD and cardiac abnormalities or symptoms (9, 59, 60), long-term studies have not consistently shown a predictive relationship between early PD after RT and serious cardiac events occurring years later (61). In the present review, when detected, heart perfusion disturbances were subclinical or failed to find a correlation with clinical outcomes. Although PD seemed to be a consequence of thoracic irradiation, no study found a link between the extent and severity of regional perfusion abnormalities and LVEF changes. With a median follow-up of 127 months (range, 19-160), Abraham et al. found that 17.2% of patients with LSBC and 5.5% of patients with RSBC had grade ≥ 2 cardiac events after RT. Cardiac events were not correlated with new PD, in contrast with the ventricular volumes receiving 5 Gy and 10 Gy (24). One limitation of this study is the use of only rest scintigraphy. Eftekhari et al. performed rest and stress MPS and found 42.9% PD at 6 months post-RT without fixed PD, meaning that a protocol without stress MPS would have found no PD (25). Radiation-induced PD imaged by MPS might not always have an immediate clinical impact, and longer follow-up may be needed.

Contradictory findings exist regarding the relationship between myocardial FDG uptake and cardiac toxicity. Some studies have shown that increased FDG uptake in the right ventricle wall is associated with cardiotoxicity after anthracycline or trastuzumab therapy (62). FDG uptake has also been predictive of clinical outcomes in other organs, such as the lung (63). In this review, two studies found that changes in cardiac SUV before and after treatment were predictive of overall survival (37, 39). While some studies found no predictive value of FDG uptake changes after RT (32), others observed a significant association with cardiac events, particularly in patients with higher MHD (38). However, the short follow-up periods and potential confounding factors, such as preexisting cardiac risk and combined treatment effects, need to be considered (64).

Despite rigorous efforts and the significant importance of the topic, the current body of research in this field has yielded largely inconclusive results. Numerous studies present contrasting findings, with only a handful suggesting any tangible correlation between nuclear medicine data and outcomes. These discrepancies underscore the pressing need for standardized methodologies, robust data collection, and a more coordinated approach to research in this domain. As it stands, the collective findings provide limited guidance for detecting early post-RT cardiac damage, a testament to the nascent and challenging nature of this research area.

## 5 Conclusion

Data suggest that incidental irradiation of the heart after breast or thoracic RT can result in early metabolic and perfusion changes. Nuclear imaging in nuclear medicine has a prominent place in the detection of these subclinical effects that could possibly predict late cardiac complications. Prospective studies with larger populations, longer follow-up, and specific heart dosimetric data are mandatory to understand the relationship between early detection of cardiac abnormalities and RIHD.

## Data availability statement

The original contributions presented in the study are included in the article/supplementary material. Further inquiries can be directed to the corresponding author.

## Author contributions

Conceptualization: JE, CB, and GN. Methodology: JE, CB, and GN. Writing: JE. Review and editing: CB, FH, CL, IC, DA, OM, and GN. Supervision: CB and GN. All authors contributed to the article and approved the submitted version.
